# Supplementary material for: Uncertainty of future projections of species distributions in mountainous regions
Source: PLoS One. 2018 Jan 10;13(1):e0189496. doi: 10.1371/journal.pone.0189496 (PMC5761832; doi:10.1371/journal.pone.0189496)
Supplement: S1 Appendix — (DOCX) [file pone.0189496.s001.docx]

**S1 Appendix. Derivation of the future projections using the delta method**

The “delta” method is the most frequently used downscaling procedure for species distribution modeling to derive future climate projections at a fine spatial scale from coarse-scale simulations from global climate models (GCMs) [1]. Three datasets are employed in the delta downscaling procedure. The first dataset consists of observations of climate variables, usually temperature and precipitation, for a recent period. Often the climate observations from individual observing stations are spatially interpolated to a regular grid, although the delta method can be applied to derive future projections at individual stations as well. The observational dataset may be a time series, typically aggregated by month or season, or may be long-term (e.g., multiple decade) averages of the climate variables. The other two datasets are simulations from a GCM for 1) a historical period and 2) a future period forced by a specific representative concentration pathway (RCP). A time slice, usually two or more decades in length, is extracted from the historical simulation. Ideally, the selected time slice overlaps, at least partially, with the time period of the observed climate dataset. Next, one or more future time slices of the same length as the time slice obtained from the historical simulation are extracted from the future simulation. Then, average monthly and/or seasonal mean, maximum, and minimum temperature and precipitation are calculated at each model grid point for both the historical and future simulations. For mean, maximum, and minimum temperature, the delta is simply the difference, at each GCM grid point, between the average (monthly or seasonal) temperature for the future time slice and that for the historical time slice. For precipitation, which is zero bounded, the delta is the ratio of the future to historical precipitation. An underlying assumption is that the deltas, since they are the difference between two model simulations, remove (at least partially) systematic biases in the GCM simulations. Because the GCM resolution is usually coarser than the spatial resolution of the gridded observed dataset, the deltas for the GCM grid points are typically spatially interpolated to the resolution of the observed dataset before they are applied to the observed values. For each climate variable, the deltas are calculated separately for each GCM/RCP/future time slice combination.

One reason for the popularity of the WorldClim dataset is that the dataset developers calculated deltas for a large number of GCMs, multiple RCPs, and several future time slices, and applied these deltas to long-term (1960-1990) monthly averages of observed mean, maximum, and minimum temperature and precipitation. The observed climate observations had initially been interpolated using a thin-plate smoothing spline algorithm that considered latitude, longitude, and elevation in addition to the values of the climate variable to a 30 second resolution (other resolutions are also available, see http://www.worldclim.org/CMIP5v1). Additional information on the downscaling procedures is available at http://www.worldclim.org/downscaling. Users can download these gridded future projections of mean, maximum, or minimum temperature and precipitation for their analyses, or, alternatively, they can download a suite a bioclimatic variables for each GCM/RCP/future time slice combination that were calculated by the WorldClim developers from the downscaled future projections of temperature and precipitation (see http://www.worldclim.org/bioclim).

Similar future projections were not available from the developers of the remotely-sensed baseline climate dataset [2]. Because our focus is on the uncertainty introduced to the future projections of species distribution from the choice of baseline (i.e., observed) climate dataset , we wanted to use the same delta values for the remotely-sensed dataset as the WorldClim developers had used for their downscaled temperature and precipitation projections to eliminate uncertainty introduced by the calculation of the deltas. To obtain the deltas employed by the WorldClim developers, we simply subtracted the 1960-1990 interpolated fields of observed monthly mean, maximum, and minimum temperature (what WorldClim refers to as the “present climate”; see <http://www.worldclim.org/version1>) from the downscaled fields of these climate variables for each GCM/RCP/future time slice combination. The interpolated present climate (i.e., observed) WorldClim fields are found at http://www.worldclim.org/current, and the downscaled future fields are available at http://www.worldclim.org/CMIP5v1. The differences are the “deltas” that the WorldClim developers applied to the observed fields. For precipitation, we calculated the ratio between the datasets to obtain the delta values used by the WorldClim developers. Next, we interpolated the observed remotely-sensed mean, maximum, and minimum temperature and precipitation fields to the same resolution of the WorldClim observed variables, and adjusted the remotely-sensed observed climate variables by the WorldClim deltas to obtain future projections of the temperature and precipitation variables. These future projections were used to calculate the 19 standard bioclimatic variables with the R package “dismo” [3].

1. Sofaer HR, Barsugli JJ, Jarnevich CS, Abatzoglou JT, Talbert MK, Miller BW, Morisette JT. Designing ecological climate change impact assessments to reflect key climatic drivers. Global Change Biology. 2017 Jul 1;23(7):2537-53.
2. Deblauwe V, Droissart V, Bose R, Sonké B, Blach-Overgaard A, Svenning JC, et al. Remotely sensed temperature and precipitation data improve species distribution modelling in the tropics. Glob Ecol Biogeogr. 2016 Apr 1;25(4):443-54.
3. Elith J, Graham CH. Do they? How do they? WHY do they differ? On finding reasons for differing performances of species distribution models. Ecography. 2009 Feb 1;32(1):66-77.
