# Supplementary material for: Uncertainty of future projections of species distributions in mountainous regions
Source: PLoS One. 2018 Jan 10;13(1):e0189496. doi: 10.1371/journal.pone.0189496 (PMC5761832; doi:10.1371/journal.pone.0189496)
Supplement: S1 Table — (DOCX) [file pone.0189496.s002.docx]

**S1­­ Table. The 21 bamboo species evaluated in this study**

| Short name | Species long name | Number of presence locations (number after removal of duplicate points within the same grid cell) |
| --- | --- | --- |
| *B. faberi* | Bashania faberi | 1636 (439) |
| *B. fargesii* | *Bashania fargesii* | 2007 (911) |
| *B. spanostachya* | *Bashania spanostachya* | 187 (45) |
| *C. szechuanensis* | *Chimonobambusa szechuanensis* | 934 (240) |
| *F. denudata* | *Fargesia denudata* | 2443 (588) |
| *F. dracocephala* | *Fargesia dracocephala* | 507 (278) |
| *F. ferax* | *Fargesia ferax Keng* | 268 (80) |
| *F. nitida* | *Fargesia nitida* | 1163(258) |
| *F. obliqua* | *Fargesia obliqua* | 191 (45) |
| *F. qinlingensis* | *Fargesia qinlinensis* | 3334 (1476) |
| *F. robusta* | *Fargesia robusta* | 745 (181) |
| *F. rufa* | *Fargesia rufa* | 967 (238) |
| *F. scabrida* | *Fargesia scrabrida* | 605 (161) |
| *P. nidularia* | *Phyllostachys nidularia* | 293 (97) |
| *Q. opienensis* | *Qiongzhuea opienensis* | 380 (99) |
| *Q. tumidinoda* | *Qiongzhuea tumidinoda* | 237 (60) |
| *Y. ailuropodina* | *Yushania ailuropodina* | 124 (38) |
| *Y. brevipaniculata* | *Yushania brevipaniculata* | 881 (243) |
| *Y. glauca* | *Yushania glauca* | 236 (61) |
| *Y. lineolata* | *Yushania lineolate* | 585 (157) |
| *Y. maculata* | *Yushania maculata* | 375 (96) |
