# Supplementary material for: Uncertainty of future projections of species distributions in mountainous regions
Source: PLoS One. 2018 Jan 10;13(1):e0189496. doi: 10.1371/journal.pone.0189496 (PMC5761832; doi:10.1371/journal.pone.0189496)
Supplement: S3 Table — The numbers 1 through 11 in the table stand for the following thresholds: “fixed cumulative value 1”, “fixed cumulative value 5”, “fixed cumulative value 10”, “minimum training presence”, “10 percentile training presence”, “equal training sensitivity and specificity”, “maximum training sensitivity plus specificity”, “equal test sensitivity and specificity”, “maximum test sensitivity plus specificity”, “balance training omission predicted area and threshold”, and “equate entropy of thresholded and original distributions”. “WC4” indicates the model calibration using the WorldClim baseline climate information and four bioclimatic variables; “RS4” refers to the model calibration using the remotely-sensed baseline climate information and four bioclimatic variables; and “RS5” refers to the model calibration using the remotely-sensed baseline climate information and five bioclimatic variables. (DOCX) [file pone.0189496.s004.docx]

**S3 Table. The true skill statistic (TSS) evaluation of model performance for the baseline climate conditions averaged over 10 replication runs for the 21 bamboo species and three model calibrations when using 11 thresholds for converting probabilities to binary species presence. The numbers 1 through 11 in the table stand for thresholds: “fixed cumulative value 1”, “fixed cumulative value 5”, “fixed cumulative value 10”, “minimum training presence”, “****10 percentile training presence”, “****equal training sensitivity and specificity”, “****maximum training sensitivity plus specificity”, “equal test sensitivity and specificity”, “maximum test sensitivity plus specificity”, “balance training omission predicted area and threshold”, and “****equate entropy of thresholded and original distributions”. “WC4” indicates the model calibration using the WorldClim baseline climate information and four bioclimatic variables; “RS4” refers to the model calibration using the remotely-sensed baseline climate information and four bioclimatic variables; and “RS5” refers to the model calibration using the remotely-sensed baseline climate information and five bioclimatic variables.**

| Threshold | 1 | 2 | 3 | 4 | 5 | 6 | 7 | 8 | 9 | 10 | 11 |
| --- | --- | --- | --- | --- | --- | --- | --- | --- | --- | --- | --- |
| Calibration | **WC4** | | | | | | | | | | |
| *B. faberi* | 0.63 | 0.67 | 0.68 | 0.59 | 0.68 | 0.66 | 0.67 | 0.66 | 0.71 | 0.62 | 0.68 |
| *B. fargesii* | 0.61 | 0.68 | 0.69 | 0.41 | 0.68 | 0.65 | 0.69 | 0.65 | 0.70 | 0.60 | 0.68 |
| *B. spanostachya* | 0.90 | 0.93 | 0.94 | 0.91 | 0.87 | 0.93 | 0.91 | 0.94 | 0.94 | 0.91 | 0.93 |
| *C. szechuanensis* | 0.82 | 0.86 | 0.85 | 0.85 | 0.80 | 0.82 | 0.86 | 0.84 | 0.87 | 0.84 | 0.86 |
| *F. denudata* | 0.73 | 0.76 | 0.76 | 0.63 | 0.75 | 0.74 | 0.77 | 0.74 | 0.78 | 0.76 | 0.77 |
| *F. dracocephala* | 0.53 | 0.57 | 0.59 | 0.53 | 0.59 | 0.53 | 0.59 | 0.54 | 0.62 | 0.53 | 0.58 |
| *F. ferax* | 0.72 | 0.84 | 0.86 | 0.85 | 0.83 | 0.86 | 0.84 | 0.84 | 0.85 | 0.84 | 0.84 |
| *F. nitida* | 0.60 | 0.65 | 0.66 | 0.57 | 0.67 | 0.66 | 0.68 | 0.66 | 0.70 | 0.61 | 0.68 |
| *F. obliqua* | 0.74 | 0.82 | 0.81 | 0.66 | 0.80 | 0.80 | 0.80 | 0.75 | 0.74 | 0.66 | 0.83 |
| *F. qinlingensis* | 0.58 | 0.62 | 0.62 | 0.47 | 0.61 | 0.54 | 0.62 | 0.55 | 0.63 | 0.56 | 0.61 |
| *F. robusta* | 0.69 | 0.83 | 0.82 | 0.75 | 0.76 | 0.76 | 0.82 | 0.79 | 0.81 | 0.81 | 0.83 |
| *F. rufa* | 0.73 | 0.82 | 0.80 | 0.71 | 0.78 | 0.79 | 0.82 | 0.80 | 0.83 | 0.82 | 0.82 |
| *F. scabrida* | 0.66 | 0.75 | 0.75 | 0.58 | 0.74 | 0.72 | 0.75 | 0.74 | 0.79 | 0.69 | 0.75 |
| *P. nidularia* | 0.77 | 0.86 | 0.86 | 0.85 | 0.81 | 0.86 | 0.85 | 0.87 | 0.86 | 0.85 | 0.85 |
| *Q. opienensis* | 0.80 | 0.85 | 0.84 | 0.84 | 0.78 | 0.82 | 0.85 | 0.83 | 0.83 | 0.83 | 0.86 |
| *Q. tumidinoda* | 0.83 | 0.91 | 0.91 | 0.91 | 0.86 | 0.91 | 0.90 | 0.88 | 0.88 | 0.87 | 0.91 |
| *Y. ailuropodina* | 0.84 | 0.86 | 0.86 | 0.89 | 0.80 | 0.87 | 0.87 | 0.80 | 0.80 | 0.85 | 0.85 |
| *Y. brevipaniculata* | 0.60 | 0.66 | 0.62 | 0.60 | 0.62 | 0.57 | 0.66 | 0.60 | 0.67 | 0.60 | 0.66 |
| *Y. glauca* | 0.82 | 0.87 | 0.86 | 0.87 | 0.79 | 0.84 | 0.87 | 0.84 | 0.84 | 0.87 | 0.88 |
| *Y. lineolata* | 0.68 | 0.71 | 0.71 | 0.70 | 0.66 | 0.63 | 0.69 | 0.64 | 0.72 | 0.69 | 0.72 |
| *Y. maculata* | 0.89 | 0.90 | 0.86 | 0.91 | 0.75 | 0.82 | 0.86 | 0.86 | 0.88 | 0.89 | 0.90 |
| Calibration | **RS4** | | | | | | | | | | |
| *B. faberi* | 0.47 | 0.62 | 0.67 | 0.50 | 0.65 | 0.62 | 0.66 | 0.64 | 0.68 | 0.56 | 0.65 |
| *B. fargesii* | 0.58 | 0.67 | 0.67 | 0.36 | 0.66 | 0.63 | 0.68 | 0.64 | 0.70 | 0.62 | 0.67 |
| *B. spanostachya* | 0.91 | 0.90 | 0.91 | 0.86 | 0.82 | 0.90 | 0.88 | 0.93 | 0.93 | 0.90 | 0.91 |
| *C. szechuanensis* | 0.80 | 0.85 | 0.84 | 0.77 | 0.80 | 0.82 | 0.85 | 0.83 | 0.86 | 0.81 | 0.85 |
| *F. denudata* | 0.60 | 0.73 | 0.75 | 0.26 | 0.73 | 0.69 | 0.74 | 0.70 | 0.76 | 0.69 | 0.73 |
| *F. dracocephala* | 0.55 | 0.62 | 0.63 | 0.53 | 0.63 | 0.62 | 0.64 | 0.62 | 0.66 | 0.57 | 0.61 |
| *F. ferax* | 0.77 | 0.79 | 0.79 | 0.08 | 0.79 | 0.78 | 0.79 | 0.79 | 0.84 | 0.79 | 0.80 |
| *F. nitida* | 0.37 | 0.62 | 0.65 | 0.48 | 0.66 | 0.62 | 0.65 | 0.65 | 0.68 | 0.55 | 0.66 |
| *F. obliqua* | 0.54 | 0.77 | 0.82 | 0.55 | 0.78 | 0.79 | 0.83 | 0.79 | 0.78 | 0.77 | 0.80 |
| *F. qinlingensis* | 0.56 | 0.60 | 0.59 | 0.53 | 0.58 | 0.50 | 0.60 | 0.51 | 0.61 | 0.55 | 0.59 |
| *F. robusta* | 0.63 | 0.80 | 0.80 | 0.66 | 0.76 | 0.77 | 0.82 | 0.77 | 0.84 | 0.77 | 0.82 |
| *F. rufa* | 0.46 | 0.67 | 0.72 | 0.41 | 0.73 | 0.73 | 0.75 | 0.74 | 0.77 | 0.64 | 0.71 |
| *F. scabrida* | 0.51 | 0.71 | 0.74 | 0.60 | 0.70 | 0.68 | 0.74 | 0.74 | 0.78 | 0.70 | 0.73 |
| *P. nidularia* | 0.52 | 0.74 | 0.80 | 0.69 | 0.78 | 0.78 | 0.78 | 0.80 | 0.82 | 0.70 | 0.80 |
| *Q. opienensis* | 0.84 | 0.86 | 0.84 | 0.86 | 0.78 | 0.82 | 0.86 | 0.85 | 0.86 | 0.86 | 0.85 |
| *Q. tumidinoda* | 0.79 | 0.89 | 0.94 | 0.90 | 0.87 | 0.90 | 0.90 | 0.90 | 0.90 | 0.87 | 0.94 |
| *Y. ailuropodina* | 0.82 | 0.83 | 0.83 | 0.82 | 0.83 | 0.85 | 0.85 | 0.83 | 0.83 | 0.81 | 0.82 |
| *Y. brevipaniculata* | 0.56 | 0.63 | 0.66 | 0.56 | 0.64 | 0.62 | 0.65 | 0.62 | 0.67 | 0.59 | 0.65 |
| *Y. glauca* | 0.65 | 0.80 | 0.85 | 0.79 | 0.78 | 0.77 | 0.84 | 0.83 | 0.84 | 0.77 | 0.84 |
| *Y. lineolata* | 0.70 | 0.76 | 0.74 | 0.69 | 0.70 | 0.68 | 0.75 | 0.73 | 0.77 | 0.74 | 0.77 |
| *Y. maculata* | 0.90 | 0.91 | 0.84 | 0.91 | 0.75 | 0.84 | 0.91 | 0.90 | 0.90 | 0.91 | 0.91 |
| Calibration | **RS5** | | | | | | | | | | |
| *B. faberi* | 0.48 | 0.63 | 0.68 | 0.51 | 0.65 | 0.61 | 0.66 | 0.62 | 0.69 | 0.59 | 0.66 |
| *B. fargesii* | 0.58 | 0.67 | 0.68 | 0.38 | 0.66 | 0.63 | 0.68 | 0.64 | 0.69 | 0.62 | 0.67 |
| *B. spanostachya* | 0.90 | 0.91 | 0.92 | 0.86 | 0.77 | 0.90 | 0.90 | 0.82 | 0.82 | 0.90 | 0.91 |
| *C. szechuanensis* | 0.80 | 0.85 | 0.84 | 0.77 | 0.79 | 0.80 | 0.84 | 0.83 | 0.86 | 0.80 | 0.85 |
| *F. denudata* | 0.61 | 0.74 | 0.74 | 0.28 | 0.72 | 0.69 | 0.75 | 0.70 | 0.76 | 0.69 | 0.73 |
| *F. dracocephala* | 0.56 | 0.61 | 0.63 | 0.56 | 0.62 | 0.59 | 0.60 | 0.62 | 0.66 | 0.57 | 0.63 |
| *F. ferax* | 0.79 | 0.82 | 0.82 | 0.08 | 0.77 | 0.80 | 0.81 | 0.80 | 0.83 | 0.80 | 0.82 |
| *F. nitida* | 0.42 | 0.66 | 0.67 | 0.52 | 0.65 | 0.64 | 0.67 | 0.66 | 0.72 | 0.66 | 0.67 |
| *F. obliqua* | 0.71 | 0.80 | 0.82 | 0.68 | 0.79 | 0.78 | 0.78 | 0.73 | 0.75 | 0.69 | 0.83 |
| *F. qinlingensis* | 0.56 | 0.60 | 0.59 | 0.53 | 0.58 | 0.50 | 0.60 | 0.52 | 0.61 | 0.55 | 0.59 |
| *F. robusta* | 0.64 | 0.81 | 0.79 | 0.68 | 0.76 | 0.77 | 0.79 | 0.77 | 0.81 | 0.75 | 0.80 |
| *F. rufa* | 0.48 | 0.71 | 0.74 | 0.53 | 0.76 | 0.74 | 0.76 | 0.75 | 0.77 | 0.69 | 0.72 |
| *F. scabrida* | 0.52 | 0.72 | 0.74 | 0.62 | 0.66 | 0.66 | 0.72 | 0.72 | 0.74 | 0.70 | 0.74 |
| *P. nidularia* | 0.52 | 0.74 | 0.78 | 0.73 | 0.74 | 0.76 | 0.79 | 0.78 | 0.80 | 0.73 | 0.79 |
| *Q. opienensis* | 0.85 | 0.84 | 0.81 | 0.88 | 0.76 | 0.82 | 0.84 | 0.86 | 0.88 | 0.87 | 0.84 |
| *Q. tumidinoda* | 0.79 | 0.89 | 0.94 | 0.91 | 0.85 | 0.90 | 0.90 | 0.90 | 0.90 | 0.87 | 0.94 |
| *Y. ailuropodina* | 0.82 | 0.84 | 0.85 | 0.83 | 0.83 | 0.86 | 0.83 | 0.80 | 0.80 | 0.81 | 0.84 |
| *Y. brevipaniculata* | 0.57 | 0.64 | 0.64 | 0.61 | 0.62 | 0.59 | 0.64 | 0.61 | 0.68 | 0.60 | 0.66 |
| *Y. glauca* | 0.67 | 0.81 | 0.85 | 0.82 | 0.79 | 0.82 | 0.83 | 0.85 | 0.86 | 0.78 | 0.84 |
| *Y. lineolata* | 0.70 | 0.75 | 0.74 | 0.68 | 0.69 | 0.67 | 0.74 | 0.72 | 0.79 | 0.75 | 0.76 |
| *Y. maculata* | 0.90 | 0.92 | 0.83 | 0.91 | 0.69 | 0.81 | 0.91 | 0.90 | 0.92 | 0.91 | 0.92 |
