# Supplementary material for: Uncertainty of future projections of species distributions in mountainous regions
Source: PLoS One. 2018 Jan 10;13(1):e0189496. doi: 10.1371/journal.pone.0189496 (PMC5761832; doi:10.1371/journal.pone.0189496)
Supplement: S4 Table — The calculations were performed using an R package available at https://github.com/vijaybarve/ENMGadgets. The calculations employed a random sample of 50% of the testing points for bootstrapping with 100 replications using thresholds for omission errors greater than 0.1, 0.5, and 0.9, respectively. “WC4” indicates the model calibration using the WorldClim baseline climate information and four bioclimatic variables; “RS4” refers to the model calibration using the remotely-sensed baseline climate information and four bioclimatic variables; and “RS5”refers to the model calibration using the remotely-sensed baseline climate information and five bioclimatic variables. (DOCX) [file pone.0189496.s005.docx]

**S4 Table. The partial area under the receiver operating characteristic curve (AUC) test results for 10 replication runs for 21 bamboo species and three calibrations. The calculations were performed using an R package available at** [**https://github.com/vijaybarve/ENMGadgets**](https://github.com/vijaybarve/ENMGadgets)**. The calculations employed a random sample of 50% of the testing points for bootstrapping with 100 replications using thresholds for omission errors greater than 0.1, 0.5, and 0.9, respectively. “WC4” indicates the model calibration using the WorldClim baseline climate information and four bioclimatic variables; “RS4” refers to the model calibration using the remotely-sensed baseline climate information and four bioclimatic variables; and “RS5” refers to the model calibration using the remotely-sensed baseline climate information and five bioclimatic variables.**

|  |  | WC4 |  |  | RS4 |  |  | RS5 |  |
| --- | --- | --- | --- | --- | --- | --- | --- | --- | --- |
|  | **Omission Error Threshold** | | | | | | | | |
|  | **0.1** | **0.5** | **0.9** | **0.1** | **0.5** | **0.9** | **0.1** | **0.5** | **0.9** |
| *B. faberi* | 1.79 | 1.76 | 1.63 | 1.78 | 1.74 | 1.57 | 1.78 | 1.75 | 1.57 |
| *B. fargesii* | 1.77 | 1.74 | 1.62 | 1.83 | 1.81 | 1.60 | 1.83 | 1.81 | 1.60 |
| *B. spanostachya* | 1.97 | 1.97 | 1.94 | 1.97 | 1.96 | 1.94 | 1.97 | 1.96 | 1.94 |
| *C. szechuanensis* | 1.91 | 1.89 | 1.84 | 1.90 | 1.89 | 1.80 | 1.90 | 1.89 | 1.81 |
| *F. denudata* | 1.85 | 1.82 | 1.73 | 1.81 | 1.79 | 1.67 | 1.81 | 1.79 | 1.68 |
| *F. dracocephala* | 1.73 | 1.70 | 1.51 | 1.75 | 1.72 | 1.56 | 1.77 | 1.73 | 1.55 |
| *F. ferax* | 1.94 | 1.94 | 1.86 | 1.91 | 1.90 | 1.79 | 1.90 | 1.89 | 1.80 |
| *F. nitida* | 1.83 | 1.81 | 1.64 | 1.80 | 1.77 | 1.58 | 1.82 | 1.80 | 1.64 |
| *F. obliqua* | 1.94 | 1.94 | 1.90 | 1.94 | 1.93 | 1.88 | 1.93 | 1.93 | 1.87 |
| *F. qinlingensis* | 1.73 | 1.68 | 1.52 | 1.65 | 1.61 | 1.49 | 1.65 | 1.61 | 1.50 |
| *F. robusta* | 1.88 | 1.87 | 1.76 | 1.89 | 1.87 | 1.76 | 1.89 | 1.87 | 1.76 |
| *F. rufa* | 1.88 | 1.86 | 1.76 | 1.84 | 1.82 | 1.67 | 1.84 | 1.82 | 1.66 |
| *F. scabrida* | 1.86 | 1.84 | 1.68 | 1.84 | 1.82 | 1.66 | 1.84 | 1.81 | 1.66 |
| *P. nidularia* | 1.92 | 1.92 | 1.80 | 1.86 | 1.85 | 1.69 | 1.85 | 1.84 | 1.69 |
| *Q. opienensis* | 1.92 | 1.91 | 1.80 | 1.93 | 1.92 | 1.85 | 1.92 | 1.92 | 1.81 |
| *Q. tumidinoda* | 1.98 | 1.97 | 1.95 | 1.97 | 1.96 | 1.93 | 1.97 | 1.96 | 1.93 |
| *Y. ailuropodina* | 1.97 | 1.97 | 1.94 | 1.95 | 1.96 | 1.92 | 1.94 | 1.94 | 1.88 |
| *Y. brevipaniculata* | 1.75 | 1.71 | 1.59 | 1.78 | 1.75 | 1.61 | 1.78 | 1.75 | 1.60 |
| *Y. glauca* | 1.95 | 1.94 | 1.90 | 1.92 | 1.90 | 1.84 | 1.93 | 1.92 | 1.86 |
| *Y. lineolata* | 1.83 | 1.81 | 1.66 | 1.87 | 1.85 | 1.71 | 1.86 | 1.85 | 1.71 |
| *Y. maculata* | 1.95 | 1.94 | 1.89 | 1.95 | 1.94 | 1.89 | 1.94 | 1.93 | 1.87 |
