# Supplementary figures and images for: Uncertainty of future projections of species distributions in mountainous regions
Source: PLoS One. 2018 Jan 10;13(1):e0189496. doi: 10.1371/journal.pone.0189496 (PMC5761832; doi:10.1371/journal.pone.0189496)

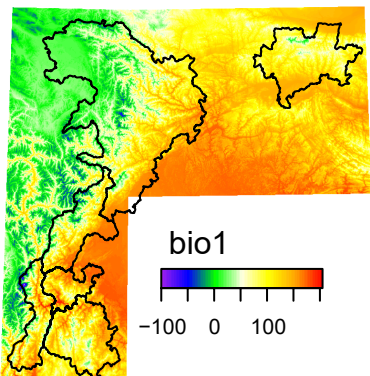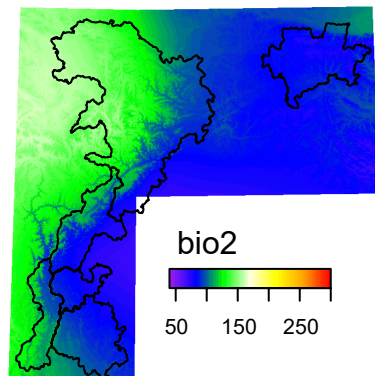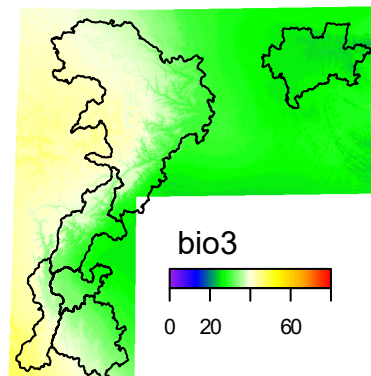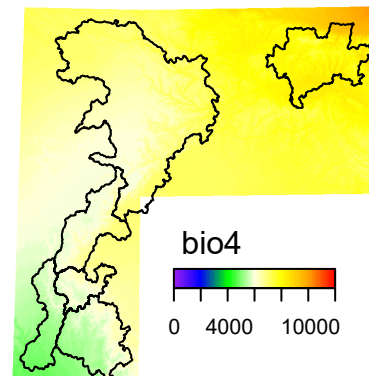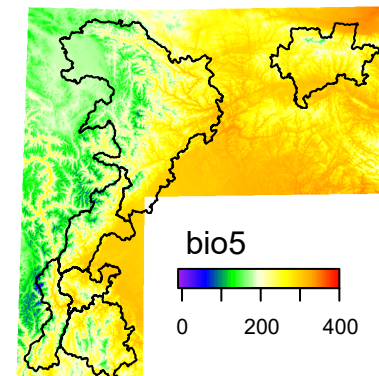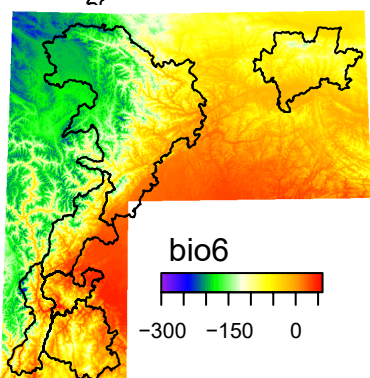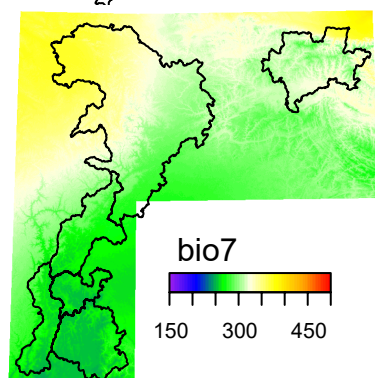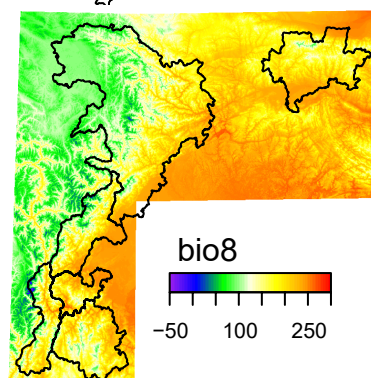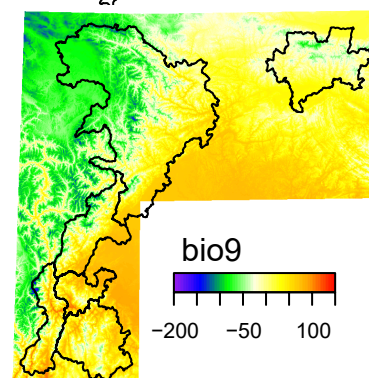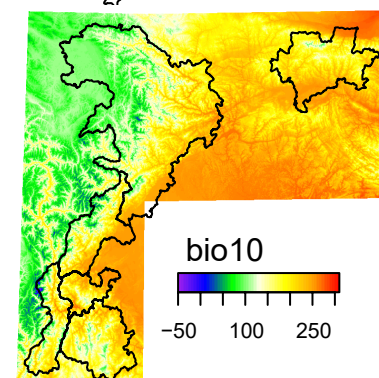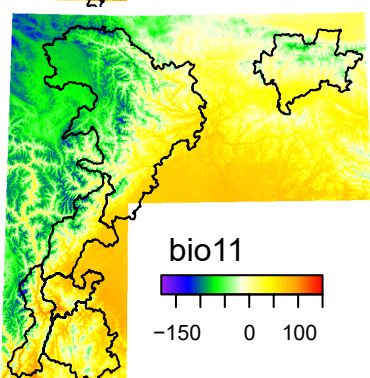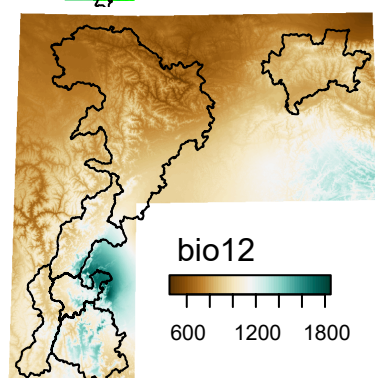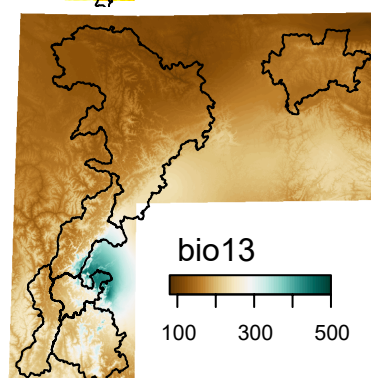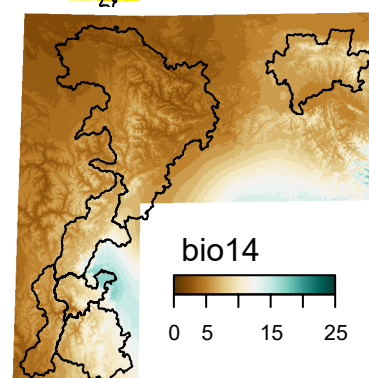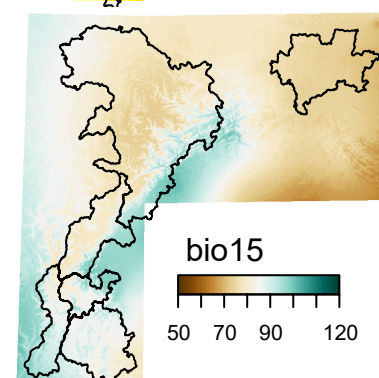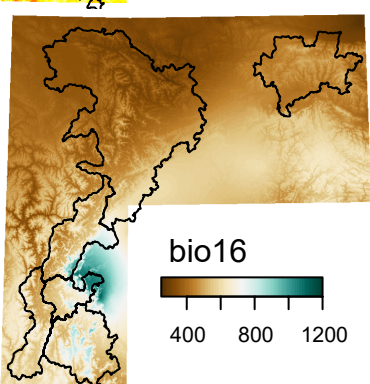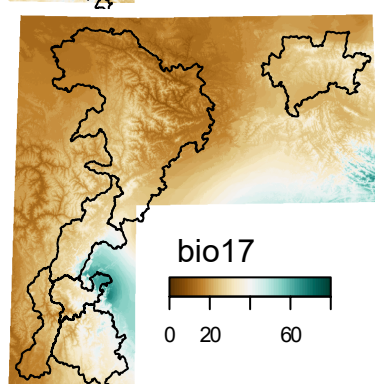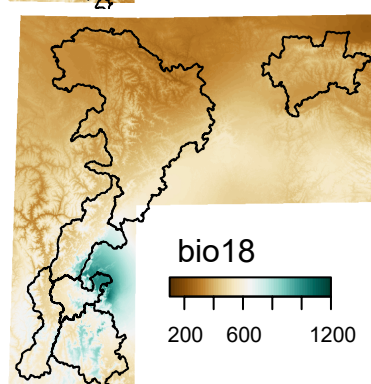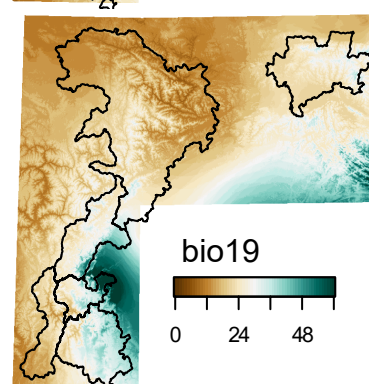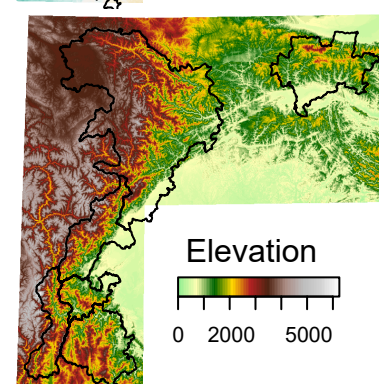

Supplement: S1 Fig — The bioclimatic variables were generated based on temperature (units of °C * 10) and precipitation (units of mm). The bioclimatic variables are long-term averages of annual mean temperature (bio1); mean diurnal range (bio2); isothermality (bio3); temperature seasonality (bio4); maximum temperature of the warmest month (bio5); minimum temperature of the coldest month (bio6); annual temperature range (bio7); mean temperature of the wettest (bio8), driest (bio9), warmest (bio10), and coldest (bio11) quarter; annual precipitation (bio12); precipitation of the wettest (bio13) and driest (bio14) month; precipitation seasonality (bio15); and precipitation of the wettest (bio16), driest (bio17), warmest (bio18) and coldest (bio19) quarter. (PDF) [file pone.0189496.s007.pdf]

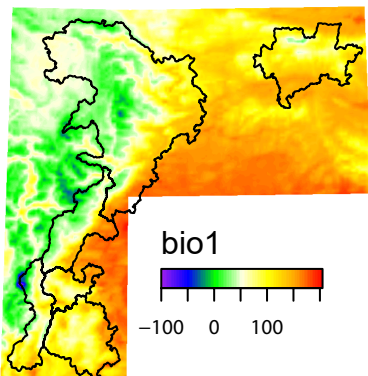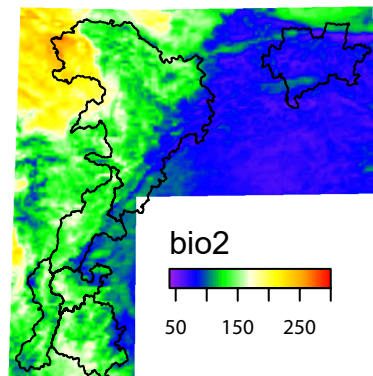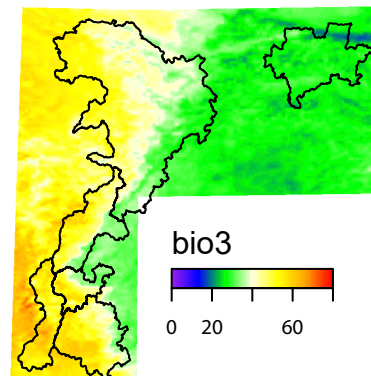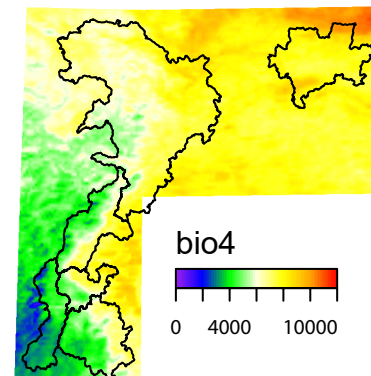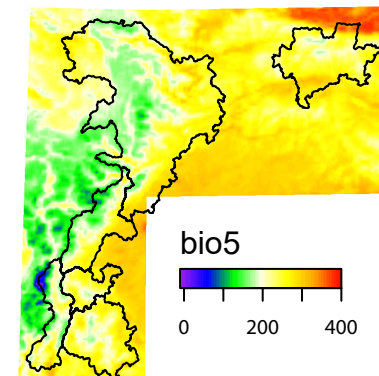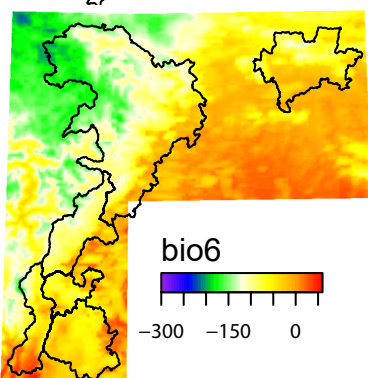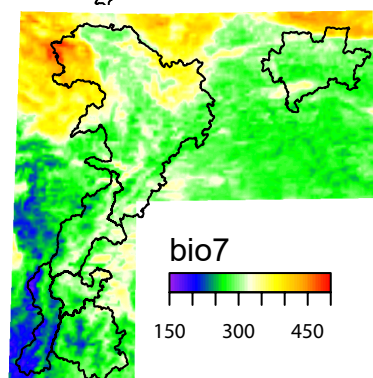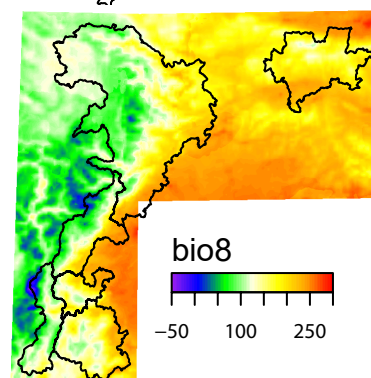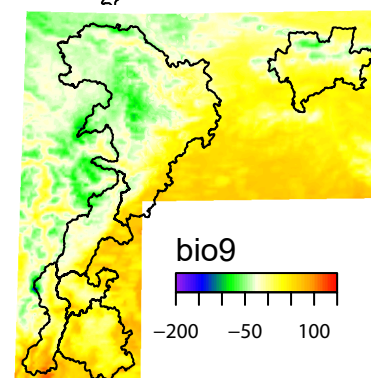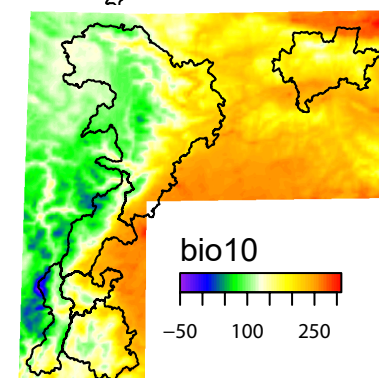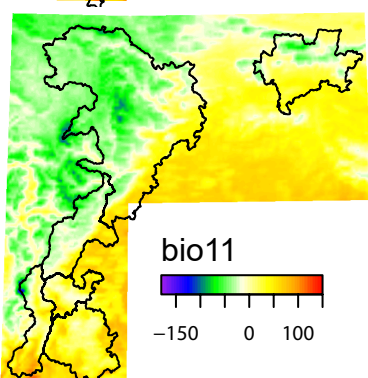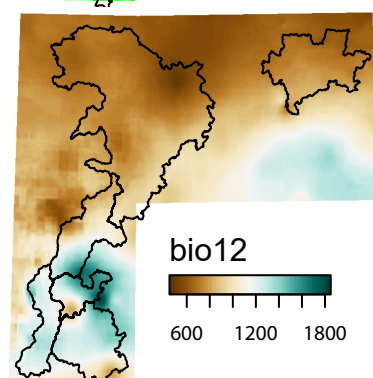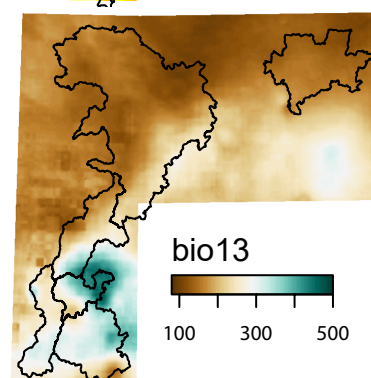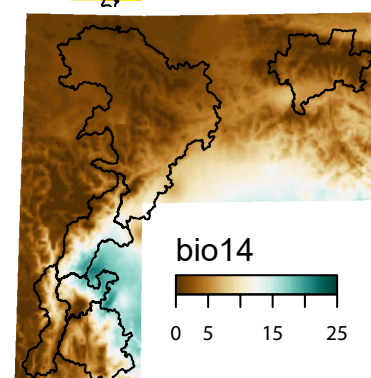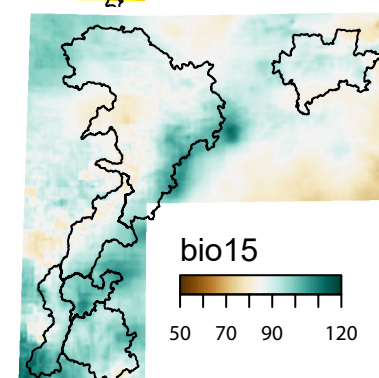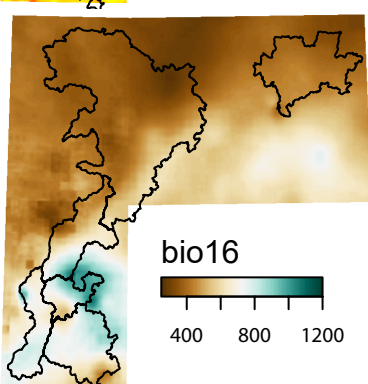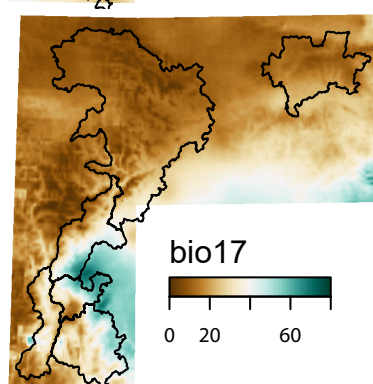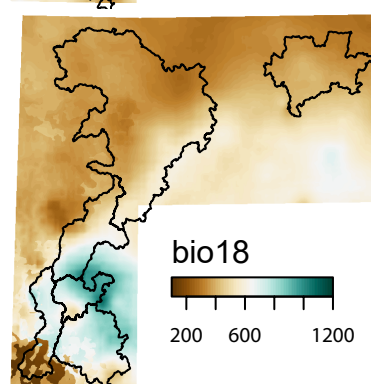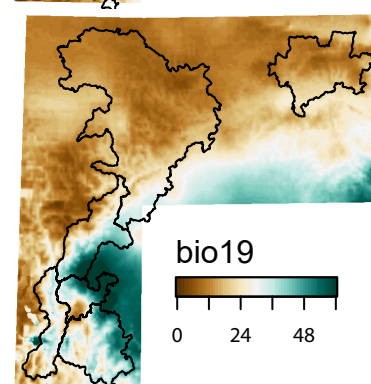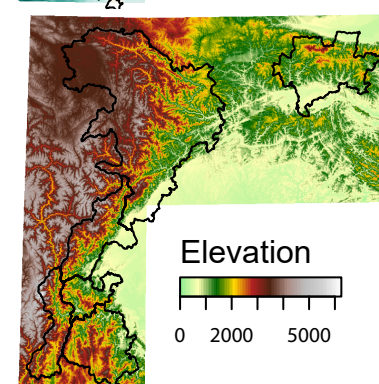

Supplement: S2 Fig — The bioclimatic variables are generated based on temperature (units of °C * 10) and precipitation (units of mm). The bioclimatic variables are long-term averages of annual mean temperature (bio1); mean diurnal range (bio2); isothermality (bio3); temperature seasonality (bio4); maximum temperature of the warmest month (bio5); minimum temperature of the coldest month (bio6); annual temperature range (bio7); mean temperature of the wettest (bio8), driest (bio9), warmest (bio10), and coldest (bio11) quarter; annual precipitation (bio12); precipitation of the wettest (bio13) and driest (bio14) month; precipitation seasonality (bio15); and precipitation of the wettest (bio16), driest (bio17), warmest (bio18) and coldest (bio19) quarter. (PDF) [file pone.0189496.s008.pdf]

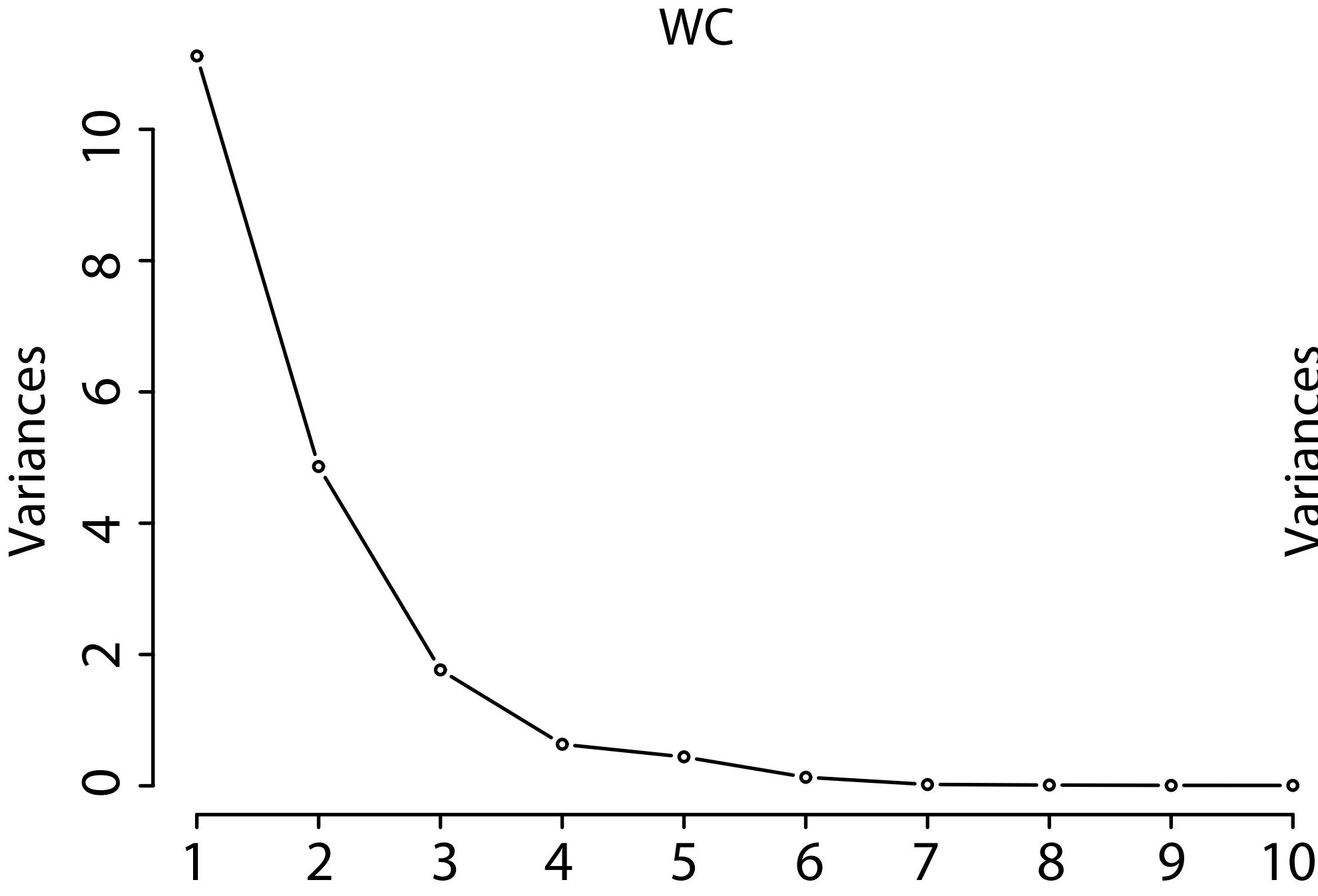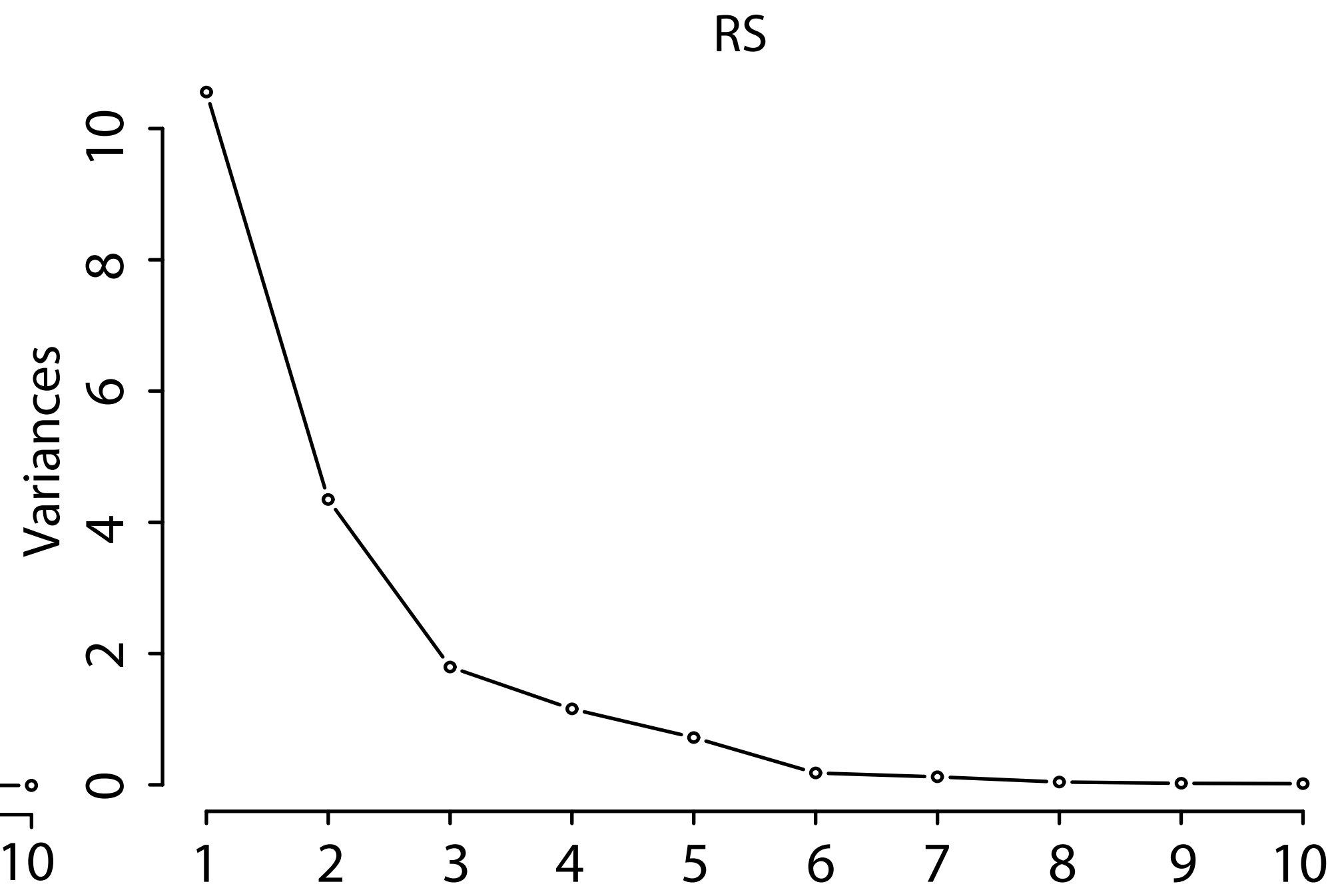

Supplement: S3 Fig — (PDF) [file pone.0189496.s009.pdf]

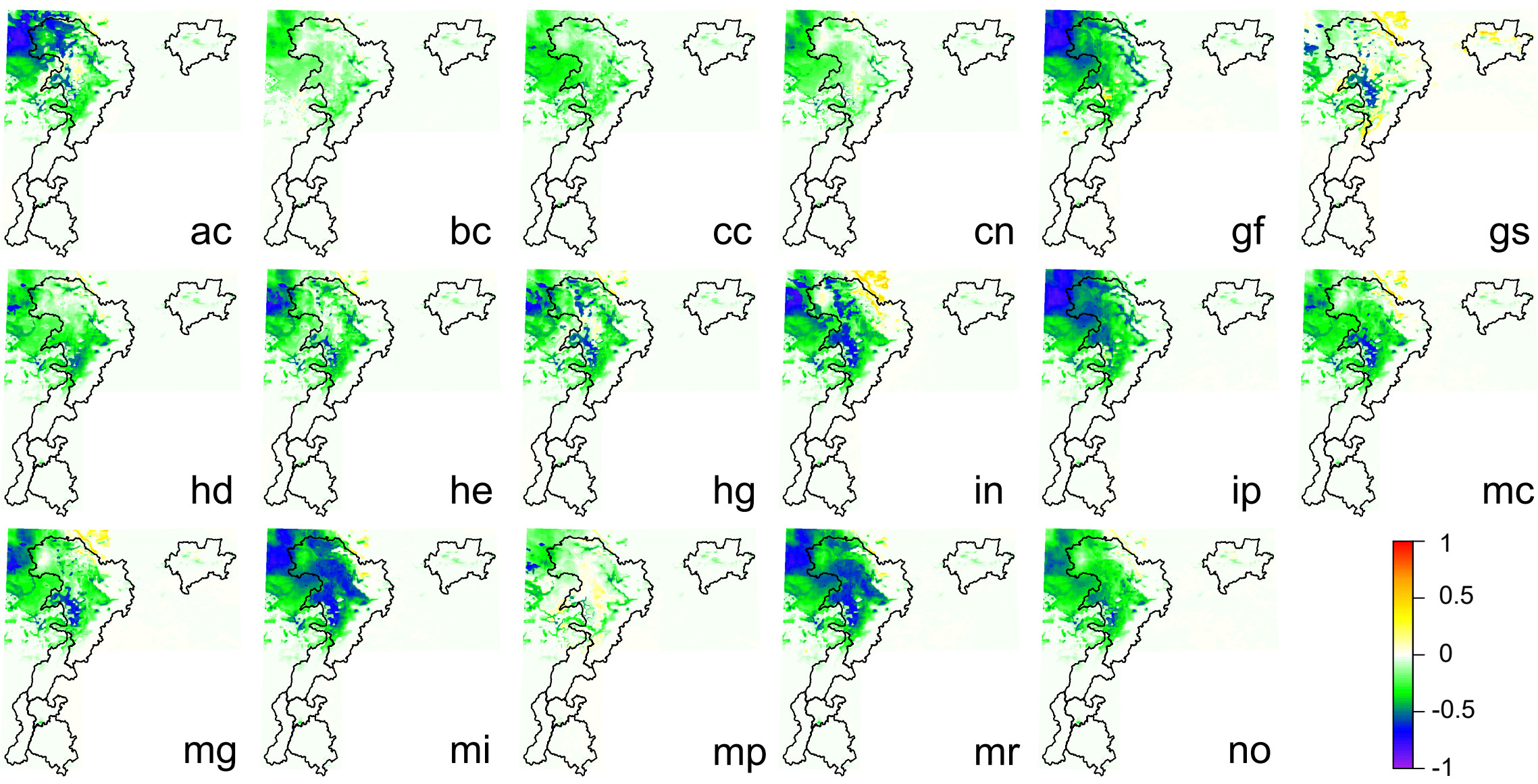

Supplement: S4 Fig — The results shown here used the “clamping” option in MaxEnt where variables outside the training range are treated as though they are at the limit of the training range. The 17 GCMs are: ACCESS1-0 (ac), BCC-CSM1-1 (bc), CCSM4 (cc), CNRM-CM5 (cn), GFDL-CM3 (gf), GISS-E2-R (gs), HadGEM2-AO (hd), HadGEM2-CC (hg), HadGEM2-ES (he), INMCM4 (in), IPSL-CM5A-LR (ip), MIROC-ESM-CHEM (mi), MIROC-ESM (mr), MIROC5 (mc), MPI-ESM-LR (mp), MRI-CGCM3 (mg), and NorESM1-M (no). (PDF) [file pone.0189496.s010.pdf]
